# Surgical treatment of monostotic fibrous dysplasia of the proximal femur in children and adolescents: Observational European Paediatric Orthopaedic Society multicenter study

**DOI:** 10.1177/18632521251355884

**Published:** 2025-07-24

**Authors:** Thomas PG van Geloven, Pieter Bas de Witte, Minna K Laitinen, Domenico A Campanacci, Kevin Döring, Dietmar Dammerer, Mohamed K Mesregah, Natasja M Appelman-Dijkstra, Mikko Haara, Giovanni Beltrami, Gerhard M Hobusch, Tanja Kraus, Sebastian Farr, Camilo Soto-Montoya, Manuel R Medellin Rincon, Javeria Saeed, Phillipp T Funovics, Lizz van der Heijden, Michiel AJ van de Sande

**Affiliations:** 1 Department of Orthopaedic Surgery, Leiden University Medical Center, Leiden, The Netherlands; 2 Pediatric Orthopaedics, Erasmus MC Sophia Children Hospital, Rotterdam, The Netherlands; 3 Bone Tumor Unit, Orthopedics and Traumatology, University of Helsinki and Helsinki University Hospital, Helsinki, Finland; 4 Orthopedic Oncology and Reconstructive Surgery, Azienda Ospedaliero Universitaria Careggi, Florence, Italy; 5 Division of Orthopedics, Department of Orthopedics and Trauma Surgery, Medical University of Vienna, Vienna, Austria; 6 Department of Orthopaedics and Traumatology, University Hospital of Krems, Krems, Austria; 7 Orthopedic Surgery, Faculty of Medicine, Menoufia University, Shebin El-Kom, Menoufia, Egypt; 8 Division of Endocrinology, Department of Medicine, Centre for Bone Quality, Leiden University Medical Center, Leiden, The Netherlands; 9 Pediatric Surgery and Orthopedics, New Children’s Hospital Helsinki, University of Helsinki and Helsinki University Hospital, Helsinki, Finland; 10 Pediatric Orthopedics Department, Azienda Ospedaliero Universitaria IRCCS A. Meyer, Florence, Italy; 11 Pediatric Orthopedic Unit, Orthopedics and Traumatology, Graz, Austria; 12 Pediatric Orthopedics and Foot and Ankle Surgery, Orthopedic Hospital Speising, Vienna, Austria; 13 Orthopedic Surgery, Instituto Nacional de Cancerologia, Bogotá, Colombia; 14 Orthopaedic Oncology/Sarcoma Unit, Fundacion CTIC Luis Carlos Sarmiento Angulo, Bogotá, Colombia; 15 Orthopedic Surgery, Aga Khan University Hospital, Karachi, Pakistan; 16 Orthopaedic Department, Princes Maxima Centre for Pediatric Oncology, Utrecht, The Netherlands

**Keywords:** Fibrous dysplasia, proximal femur, child and adolescent, watchful waiting, internal fixators

## Abstract

**Purpose:**

Monostotic fibrous dysplasia is a rare benign fibro-osseous disorder. Proximal femoral monostotic fibrous dysplasia is especially vulnerable to pathological fracture and deformation, requiring specific treatment strategies. Literature on pediatric proximal femoral monostotic fibrous dysplasias is sparse and without consensus. We present the largest observational cohort study on various treatment methods of pediatric proximal femoral monostotic fibrous dysplasia.

**Methods:**

Pediatric patients with proximal femoral monostotic fibrous dysplasia were included, from 10 academic hospitals for oncological orthopedics (2000–2021). Baseline characteristics, treatment strategies, and complications were assessed. Primary outcomes were failure rates, failure-free survival, and risk factors for failure. Failure was defined as fracture, progressive deformity, or surgical (re-)intervention after the start of treatment.

**Results:**

Forty-one pediatric patients with proximal femoral monostotic fibrous dysplasia were included (median age = 11 years (range = 6–16), *n* = 21 (51%) male). Median follow-up was 5.1 years (range = 0.8–18.6). Index procedure was watchful waiting (*n* = 9), percutaneous procedure (*n* = 4), open procedure (*n* = 15), or internal fixation (*n* = 13). Failure rates were 11%, 50%, 40%, and 31%, respectively (*p* = 0.41). Overall, 2- and 5-year failure-free survival was stable at 87.5% (95% confidence interval = 64.6–110.4). Risk factors associated with failure were fracture at diagnosis (hazard ratio = 3.7, 95% confidence interval = 1.2–11.5), calcar involvement (hazard ratio = 2.6, 95% confidence interval = 0.7–9.4), and male sex (hazard ratio = 2.1, 95% confidence interval = 0.6–7.8).

**Conclusion:**

In cases with low fracture and deformity risk, watchful waiting can be a viable management option for proximal femoral monostotic fibrous dysplasia. When intervention is necessary, internal fixation is advised to prevent fractures and deformity. Curettage with grafting or bone substitute injections should be used with hesitance. Currently, there is no clearly superior treatment for pediatric proximal femoral monostotic fibrous dysplasia, leaving treatment choices to be based on individual characteristics.

## Introduction

Fibrous dysplasia (FD) is a benign genetic disorder that forms medullary, fibro-osseous lesions causing focal inadequate and poorly organized mineralized bone, replaced by fibro-osseous tissue.^1^ This tissue can contain cystic components that may span almost the entirety of the lesion,^1^ in which case we call it cystic degenerated FD. FD is caused by a somatic mosaic missense mutation in the GNAS1 gene, which can also be present outside the bone. When skeletal lesions are combined with extraskeletal manifestations, the disease is referred to as McCune–Albright syndrome (MAS). Since features of the disease will not present at the same time, we now refer to the condition as FD/MAS. FD/MAS is mostly diagnosed during childhood (between ages 11 and 20) but can remain asymptomatic into adulthood.^2,3^ Presenting symptoms might be pain, altered gait, fractures, and deformities.^2-4^ FD lesions can present in one bone (monostotic) or in multiple bones (polyostotic). The monostotic form is 1.5–2.5 times more common than the polyostotic variant,^3^ and generally has a milder course, with less tendency to cause deformity.^5,6^

Treatment can be pharmacological and/or surgical and should be focused on maintaining function, pain reduction, and structural stability for the pediatric population to ensure unhindered daily activities as much as possible. Surgical treatment strategies for (cystic degenerated) FD of the proximal femur include internal fixation, mainly with plates or intramedullary nailing,^7–9^ or curettage with strut graft. In the past, cancellous bone grafting was often used; however, due to resorption of the cancellous bone, this is now mostly abandoned.^2,10^ Cortical strut grafts have been used for proximal femoral FD, although Majoor et al. reported a 46% revision rate, and 29% resorption rate.^11^ In case of cystic degeneration, percutaneous procedures with injections are a possibility.

Treating FD in the proximal femur may present difficulties. Due to the high (micro)fracture rate in this weight-bearing location,^12^ causing deformity and ultimately the characteristic shepherd’s crook deformity, it is deemed the most problematic anatomic location.^10^ Currently, due to the rarity of the disease, most research has been conducted on a small scale,^5,7,13,14^ not specifically for monostotic FD,^5,6^ or has not specifically targeted children and adolescents,^8^ even though this group is generally recognized as having the most aggressive disease course with the highest risk of symptoms, deformation, and/or fracture.^4,6,11^

We present the largest cohort of pediatric proximal femoral monostotic FD patients to date, through an international multicenter cooperative study group. We evaluated outcomes and failure-free survival (FFS) of different treatment methods and watchful waiting, and assessed potential risk factors for failure. We aimed at finding associations between monostotic FD (MFD) characteristics and treatment failures, enabling clinicians to make informed decisions for individual MFD lesions in this weight-bearing location.

## Methods

In this international cooperative study instituted by the (institution), members of the European Paediatric Orthopaedic Society (EPOS), European Musculo-Skeletal Oncology Society, and International Society of Limb Salvage have collaborated and contributed. All suitable pediatric patients with (cystic degenerated) MFD in the proximal femur, treated in 10 participating academic hospitals for oncological orthopedics across 4 continents between 2000 and 2021, were included. This study was part of a larger cooperative project to study the treatment of bone cysts.^15,16^

Patients aged ≤16 years at the time of diagnosis of proximal femoral monostotic FD and a minimal follow-up of 6 months were included. Diagnosis was confirmed by roentgenography, magnetic resonance imaging (MRI), and/or histology. Exclusion criteria were polyostotic FD/MAS/Mazabraud syndrome, as we consider these a significantly different patient group with typically a more severe clinical course and more aggressive (systemic) treatment.

Individual centers collected data from medical charts, which were stored in a pseudonymized database. Medical records were analyzed for demographics, diagnostic features, and treatment characteristics, complications and reinterventions during follow-up.

Index procedures were classified as watchful waiting, percutaneous treatment (sclerotherapy or filling with injectable bone substitute), open surgical treatment (curettage with or without adjuvants/filling/internal fixation), or internal fixation alone (i.e., without treatment targeting the FD lesion). Cyst dimensions, estimated cyst volume, and neck–shaft angle were measured on plain radiographs by experienced orthopedic surgeons in each center following standardized methods (Supplement 1) at diagnosis and last follow-up. The estimated cyst volume (cm^3^) was approximated centrally using the formula for ellipsoid volume: *V* = $\frac{4}{3}\times \pi \times r_{\mathrm{AP}}\times r_{\mathrm{CC}}\times r_{\mathrm{ML}},$ as this most closely resembles the true volume of cysts. The radi (r) were determined by halving maximal anteroposterior (AP) × craniocaudal (CC) × mediolateral (ML)) in mm. Width was estimated by measuring the maximal ML distance in mm (Supplement 1). Location was defined as epiphysis if the lesion was cranially from the physis, metaphysis if the lesion was cranially from the lesser trochanter, meta-diaphysis if the lesion was located in both metaphysis and diaphysis, and diaphysis if the lesion was located distal to the lesser trochanter, not surpassing the isthmus.

Outcome measures were 2- and 5-year FFS, number of percutaneous or open re-procedures performed (due to progression of pain, deformity, or (impending) fractures), and complications. For watchful waiting, failure constituted a pathologic fracture, progressive deformity indicating fixation or corrective osteotomy, or any surgical or percutaneous intervention during follow-up. For all other index procedures, failure was defined as pathologic fracture, progressive deformity indicating fixation or corrective osteotomy, or surgical (re-)intervention during follow-up.

According to our Institutional Review Board and Dutch law, this observational cohort study was not subject to the Medical Research Involving Human Subjects Act (G19-064) and need for patient informed consent was waived. Each collaborating center handled informed consent according to its local legislation.

### Statistical analysis

Baseline descriptive analyses for the entire group were stratified for index procedure. Continuous data had a non-normal distribution and was described using medians and ranges. Categorical variables were depicted with frequencies and percentages (%).

Comparisons for failure rates between index procedures were made using chi-squared tests. For survival analyses, FFS was determined from the index procedure until failure, by Kaplan–Meier graph with log-rank tests. FFS was represented for the 2- and 5-year timepoints in percentages with 95% confidence interval (95% CI).

Risk factors for failure were estimated using univariable Cox-regression analyses. Included covariables were sex, age <10 years at diagnosis, fracture at diagnosis, cyst volume >33 cm^3^, and involvement of the calcar femorale. These factors were selected based on existing literature. Sex was studied in prior research, but has so far not been marked as risk factor for failure.^4^ Young age was previously hypothesized as a risk factor due to a more aggressive nature.^4,6,11^ Fractures preceding treatment were identified as risk factor for needing revision surgery and failure.^11^ Involvement of larger percentage of the femur and higher radiolucency were also associated with deformity.^6^ Since previous literature did not cite a cutoff point for volume, median cyst volume at diagnosis was used, which was 33 cm^3^. Increased deformity and fracture rates have been described for involvement of the calcar femorale, due to loss of stress force redistribution.

Estimated hazard ratios (HRs) and 95% CIs were reported.

No imputation methods were used on missing data. IBM Statistical Package for Social Statistics (SPSS) version 29 (Chicago, IL, USA) was used for analysis.

## Results

Out of 47 pediatric patients with MFD of the proximal femur, 41 were included. Five patients were excluded because of previous treatment elsewhere before referral. One patient was excluded because of insufficient follow-up (4 months). Baseline characteristics were similar for included and excluded patients.

Baseline characteristics are depicted in Table 1. There were some missing data on the following: distance to physis (*n* = 1), neck–shaft angle at diagnosis and last follow-up (*n* = 2), and cyst volume at diagnosis and minimal cortical thickness (*n* = 3). Median age at diagnosis was 11 years (range = 5–16), and 51% (*n* = 21) were male. Median follow-up was 5.2 years (range = 0.8–18.6).

**Table 1.** Patient characteristics at baseline.

	Watchful waiting	Percutaneous procedure	Open procedure	Internal fixation alone	Total group
Group size	9	4	15	13	41
Demographics					
Male, *n* (%)	4 (44.4)	2 (50)	10 (66.7)	5 (38.5)	21 (51.2)
Age at diagnosis, median (range)	14 (7–16)	13 (11–15.6)	11 (5–16)	10 (5–16)	11 (5–16)
Follow-up, median (range)	4.7 (0.8–13)	8.4 (1.4–15.7)	4.1 (1.1–18.6)	5.2 (1.1–13.6)	5.2 (0.8–18.6)
Localization					
Epiphysis, *n* (%)	0	0	2 (13.3)	2 (15.4)	4 (9.8)
Metaphysis, *n* (%)	5 (44.4)	2 (50)	5 (33.3)	2 (15.4)	14 (34.1)
Meta-diaphysis, *n* (%)	4 (55.6)	2 (50)	6 (40)	8 (61.5)	20 (48.8)
Diaphysis, *n* (%)	0	0	2 (13.3)	1 (7.7)	3 (7.3)
Size					
Width, median (range)	40 (23–70)	34 (30–50)	33 (16–89)	45 (23–70)	40 (16–89)
Estimated volume, median (range)	38 (24–59)	33 (16–53)	36 (4–273)	26 (8–128)	33 (4–273)
Closest distance to physis, median (range)	27 (4–56)	17 (10–58)	12 (0–57)	17 (0–70)	18.5 (0–70)
Minimal cortical thickness, median (range)	3 (1–4)	21 (1–2.7)	1 (0.3–3)	1 (1–2.1)	1.25 (0.3–4)
Involvement of calcar femorale, *n* (%)	3 (33.3)	3 (75)	10 (66.7)	8 (72.7)	24 (58.5)
Fracture at diagnosis, *n* (%)	2 (22.2)	1 (25)	3 (20)	4 (30.7)	10 (24.4)
Neck–shaft angle at diagnosis, median (range)	134 (126–154)	134 (129–142)	139 (114–161)	135 (66–141)	135 (66–161)

Age at diagnosis and follow-up were presented in years, volume in cm^3^, distance to physis and cortical thickness in mm, and neck–shaft angle in degrees.

Diagnosis was confirmed by MRI in 30 patients (73%). Twenty biopsies (49%) were performed. In addition, computed tomography (*n* = 17), bone scintigraphy (*n* = 16), and plain radiographs (*n* = 14) were performed to determine diagnosis.

**Figure 1.**
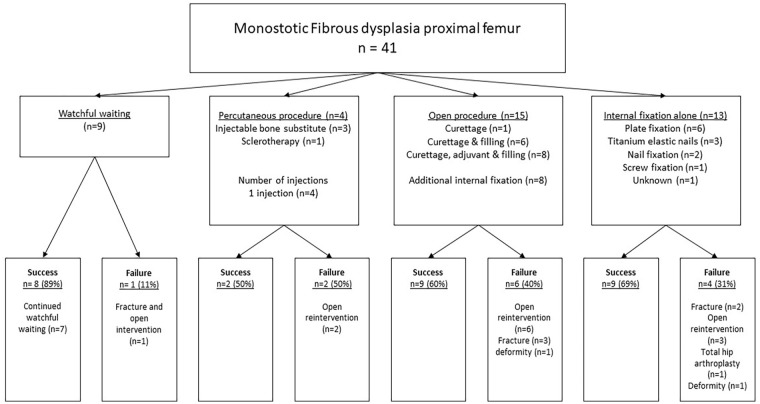
Flowchart of the index procedures and their successes and failures.

Index procedures were watchful waiting (*n* = 9), percutaneous treatment (*n* = 4), open surgery (*n* = 15), or internal fixation alone (*n* = 13; Table 2 and Figure 1). Open (re-)procedures after index procedure were conducted 21 times throughout the follow-up period. The number of patients who underwent open surgery for (impending) fractures after index procedure in each group was 1/9 (11%) watchful waiting, 2/4 (50%) percutaneous, 6/15 (40%) open procedure, and 3/13 (23%) for internal fixation alone (*p* = 0.34).

**Table 2.** Index procedures.

	Fibrous dysplasia (*n* = 41)
Watchful waiting	9 (22.0%)
Percutaneous procedure	4 (9.8%)
Injectable bone substitute	3 (7.3%)
Sclerotherapy	1 (2.4%)
Open procedure	15 (36.6%)
Curettage	1 (2.4%)
Curettage and filling	6 (14.6%)
Curettage and adjuvants and filling	8 (19.5%)
Additional internal fixation	8 (19.5%)
Pediatric hip plate	2 (4.9%)
Angled blade plate	4 (9.8%)
Titanium elastic nails	2 (4.9%)
Internal fixation alone	13 (31.7%)
Pediatric hip plate	5 (12.2%)
Angled blade plate	1 (2.4%)
Titanium elastic nails	3 (7.3%)
Intramedullary nail	2 (4.9%)
Screw	1 (2.4%)
Unknown	1 (2.4%)
Other additional procedures in the same setting	
Hardware removal	2 (4.9%)
Epiphysiodesis	1 (2.4%)
No. of open re-procedures in follow-up	
0	27 (65.9%)
1	9 (22.0%)
2	3 (7.3%)
3 or more	2 (4.9%)
Complications	
Infection	0
Fracture	6 (14.6%)
Deformity	2 (4.9%)

Fractures were the main complication during follow-up. Six fractures were seen after index procedure: one after 1.8 years of watchful waiting (8.8 years old), two after titanium elastic nails (TENs; 13.6 and 13.2 years old, after 6.6 and 1.2 years, respectively), and three after curettage and filling with bone graft, of whom two had additional internal fixation (14.3, 15.4, and 16 years old, after 2.3, 8.4, and 0.3 years, respectively).

Two varus neck deformities were observed during follow-up. The first 9 months after open procedure, and the second one, originally treated with TENs and had a pathological fracture after 6.8 years of follow-up. The median neck–shaft angle of the entire cohort stayed stable at 135° (range at diagnosis = 66–161, at last follow-up = 127–153).

**Figure 2.**
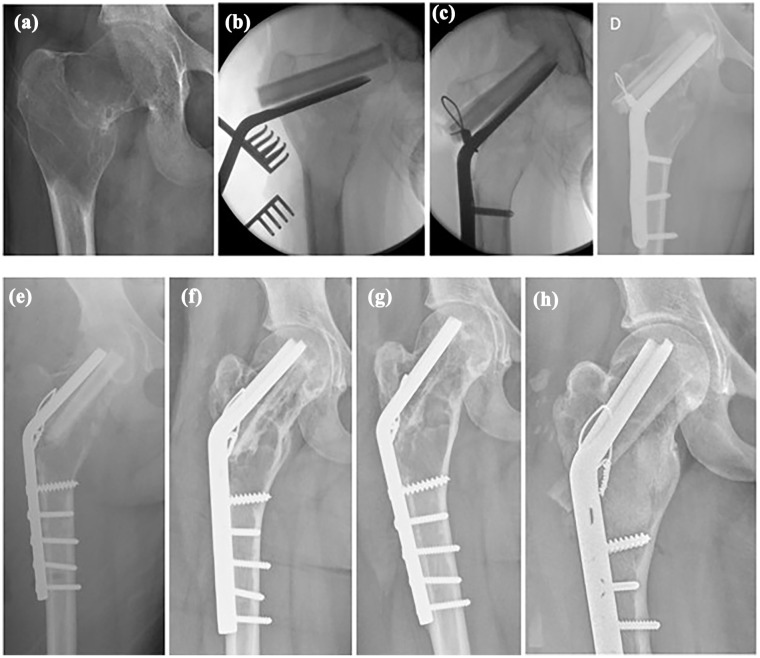
A 15-year-old girl presenting with pain, (a) on radiographs cystic degenerated monostotic fibrous dysplasia in the right proximal femur with neck–shaft angle of 113°. (b) Intraoperative imaging, 120° angled blade plate with two fibular strut grafts places cranially. (c) Pulling screws brought the distal part of the plate toward the femur shaft, while lifting the femoral neck into a normal neck–shaft angle, without osteotomy. Cerclage was conducted for added stability. (d) One day postoperative, there was protrusion of the angled blade plate into the intra-articular space due to soft bone. (e) Revision, 2 days post-original operation with shorter 130° blade plate and more distal screws. (f) After 8 months, the consolidation of the cyst with resorption of the strut graft. (g) One year post-operation, the strut graft is fully resorbed, but there is thickening of the cortices. (h) Last follow-up, patient returned after 6 years and 3 months with pain and abnormal gait, for which new cortical strut grafts were placed along with Vitoss synthetic cancellous bone filling. After this, there were no more symptoms.

Other complications were osteosynthetic material breakout (*n* = 1) and severe osteoarthritis (*n* = 1). The material breakout was observed for a 125° angled blade plate 2 days after initial surgery, probably due to low bone density, despite pre-emptive cerclage and strut graft placement. A revision with a 130° plate and fibular strut grafts was done 2 days later (Figure 2). Severe osteoarthritis was seen in a 21-year-old patient, 12.6 years after index procedure (plate fixation alone), eventually requiring total hip arthroplasty.

No infections were seen during follow-up.

One patient was treated with polidocanol (Aethoxysklerol) injection, leading to approximately one-third cyst size reduction and alleviation of symptoms during follow-up.

Failure rates were as follows: watchful waiting 1/9 (11%), percutaneous procedures 2/4 (50%, both injectable bone substitute), open procedures 6/15 (40%, two with additional pediatric hip plate as internal fixation, and four without), and internal fixation alone 4/13 (31%, two with pediatric hip plate, two with TENs; *p* = 0.41; Figure 1). The group that received internal fixation (with or without additional curettage) had an overall failure rate of 6/21 (28.6%) compared to 7/20 (35%) in the group without fixation (*p* = 0.66). Failures were seen in 0/1 DHS, 4/12 (33%; angled blade) plates, 2/5 (40%) TENs, 0/2 intramedullary nail fixation, and 0/1 undefined osteosynthesis (*p* = 0.93).

**Figure 3.**
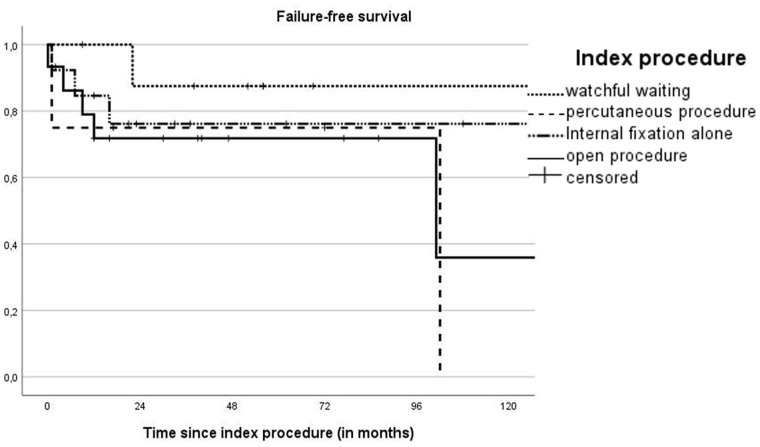
Kaplan–Meier curve of FFS including numbers at risk and cumulative incidence of failure (Cum. events) at different timepoints. FFS: failure-free survival.

Overall, 2- and 5-year FFS rates were stable at 76.8% (95% CI = 63.5–90.1). For watchful waiting, percutaneous procedures, open procedures, and internal fixation alone, FFS was stable at the 2- and 5-year interval at 87.5% (95% CI = 64.6–110.4), 75.0% (95% CI = 32.5–117.5), 76.2% (95% CI = 52.5–99.9), and 71.8% (95% CI = 48.3–95.3), respectively (*p* = 0.35; Figure 3).

Univariable HR values for failure were as follows: pathological fracture at diagnosis (HR = 3.7, 95% CI = 1.19–11.49), involvement of the calcar (HR = 2.60, 95% CI = 0.71–9.43), male sex (HR = 2.17, 95% CI = 0.58–7.81), volume >33 cm^3^ (HR = 1.01, 95% CI = 0.32–3.19), and age <10 years (HR = 0.97, 95% CI = 0.29–3.22; Table 3).

**Table 3.** Potential individual risk factors for failure, estimated using univariate Cox regression model.

	*N* (total)	Failure (total)	Hazard ratio	95% Confidence interval
Fracture at diagnosis	10	6	3.70	1.19–11.49
N/A				
Involvement of calcar	24	10	2.60	0.71–9.43
N/A				
Male	21	7	2.13	0.58–7.81
Female				
Volume >33 cm^3^	20	6	1.01	0.32–3.19
<33 cm^3^				
Age at diagnosis <10 years	13	6	0.97	0.29–3.22
>10 years				

## Discussion

The aim of this international multicenter study on the treatment of MFD in the proximal femur of children was to evaluate various treatment strategies for clinical outcomes, FFS, and potential risk factors for failure. As such, we strived to find associations between cyst characteristics and treatment failure in this weight-bearing location, enabling clinicians to make well-informed, individualized treatment choices.

Watchful waiting showed to be a feasible management strategy in selected cases of proximal femoral MFD without pain and with pre-existing low fracture and deformity risk. If treatment is required, intramedullary fixation is advised to prevent fractures and deformity. Presentation with a pathological fracture at the time of diagnosis demonstrated to be a risk factor for failure.

### Failure and risk factors for failure

Failure rates in this study were comparable between treatment groups. More failures were seen in patients with fracture at diagnosis.^4^ This may be explained by already poor bone quality and structural integrity in these patients at diagnosis, resulting in fracture. The continuous replacement of bone, even after fracture, might result in maintained reduced bone strength without the self-healing properties as observed in, for example, simple bone cysts.^15^

Involvement of the calcar femorale showed a possible association with failure, as biomechanically, the loss of stress force redistribution in the proximal femur results in increased deformity and fracture risk.^5,6,11,17^

Age and cyst volume were not significantly related to failure in our study.^4,18^ For age, a further subdivision does not seem relevant for failure other than child versus adult. For cyst volume, as per indication bias, the largest cysts (all those above 60 cm^3^) received more aggressive treatment, which may have contributed to their low failure rate.

### Surgical treatment

The treatment of choice in this location has differed considerably over the years. Curettage with bone grafting was historically the operation of choice. However, reports of a high conversion rate of cancellous bone grafts into fibrous tissue have, in recent literature, led to a consensus defining it as an ineffective treatment.^8,19^ The application of cortical strut grafts, however, has been anchored in the current best practice management guideline for treatment of symptomatic proximal femoral FD without varus deformation, shaft involvement, and history of fracture, and with good bone stock in the proximal femoral neck.^19^ However, the reported 46% revision rates and 29% resorption rate in available literature,^11^ combined with our 50% revision rate and resorption rate is in our opinion quite high, posing concerns about the efficacy of this treatment.

The use of internal fixation did not result in a significant reduction in failure rates in our study, although this can be attributed to confounding by indication. When examining the individual fixation types, TENs had a 2/5 failure rate, as TENs do not prevent progression, deformity, or subsequent fractures in children with MFD.^2,8,20^ In addition, due to impaired bone quality, the cortices may be too thin to properly guide and contain TENs.^21^ Also (angled blade) plates had a 4/12 failure rate, although these are mostly deployed in cases when intramedullary nailing is not possible, such as deformity, or in children which are too small for regular intramedullary nailing. Intramedullary nailing or plate osteosyntheses are advised, ideally personalized to the individual proximal femur, that due to deformity and impaired bone quality may have an increased risk of material breakout (Figure 2). Our study suggests that internal fixation alone is a good option for managing fracture and deformity risk in MFD in the proximal femur, supported by a previous study of Ebeid et al. where they performed internal fixation, preventing fracture, and deformity in 17/18 monostotic lesions.^7^ The best form of fixation is still up for debate, although intramedullary fixation, if feasible, has some advantages over extramedullary, such as lower morbidity, better biomechanics, and coverage of a longer trajectory.^22^

### Watchful waiting

The goal of watchful waiting is to follow asymptomatic patients radiographically at least until skeletal maturity, after which monostotic lesions typically stabilize, and avoid surgical morbidity whenever possible. Watchful waiting strategies are often used in cases of craniofacial FD,^19,23^ yet are generally not recommended in the proximal femur due to higher fracture and deformity risks.^10^ However, one needs to take into account that published cohorts often include more severely affected populations with polyostotic FD and MAS, which might overestimate the complication rate.^4,6,12,24^

Our watchful waiting proximal femoral MFD subgroup however, had an 89% success rate, which agrees with 85% success rate in proximal femoral MFD in all ages by Han et al.^18^ This indicates that in selected lesions, watchful waiting is a viable option, in concordance with Stanton et al.^9^ and Soveral Pereira et al.^22^

With our watchful waiting group having comparatively thick cortices, few lesions involving the calcar femorale, slightly higher ages, and only involvement of the metaphysis and meta-diaphysis, these results cannot be projected onto all patients with MFD in the proximal femur, due to confounding by indication. Therefore, it should not be regarded as universal index procedure for proximal femoral MFD. Eligible patients should be asymptomatic (no pain/limping) and preferably have a low risk of fracture and deformity, with no risk factors for failure: no (micro)fracture at diagnosis, no calcar involvement, normal neck–shaft angle, and moderate size of (cystic) FD.

### Minimally invasive percutaneous procedures

Injectable bone substitute theoretically would represent an excellent treatment option in cystic degenerated MFD, adding mechanical stability to the lesion with injections only. However, as the failure of 2/3 bone substitute injections in our study shows, it does not seem to prevent progression and the need for eventual open reintervention. Also, according to DiCaprio et al.,^25^ the added mechanical stability is moderate, while being resorbed like all other bone grafts.

The use of polidocanol in FD has, to the best of the authors’ knowledge, only been described by Majoor et al.^26^ In our study, we saw that our patient with polidocanol had a one-third cyst size reduction. A possible explanation for the mechanism is stimulation of bone remodeling, decreasing cyst size. However, the underlying FD lesion will persist, as the injection does not alter the GNAS1 mutation.

### Strengths and limitations

The advantage of this study lies in the specificity and relatively large size of the cohort, made possible by its international multicenter character. As FD is an incredibly heterogeneous entity, treatments are not universally translatable to all forms and locations, especially to the proximal femur with considerable risk of deformity and fracture. In addition, due to the late average presentation age, most MFD studies typically contain adults, who on average have less active disease.^1^ Therefore this study supports and advances previous studies with smaller cohorts and treatment in all ages.^7,13,14,18,27^ Also, the wide array of treatment modalities studied offers an excellent overview of current clinical practice and supports watchful waiting in MFD of the proximal femur.

The main limitation of this study lies in its observational nature, inherently producing confounding by indication, and due to the small subgroups, correction by multivariable regression models could not be performed. Second, the relatively short minimal follow-up time is not truly representative of this patient group, as it is at risk of failure even after skeletal maturity. Third, the diagnosis of MFD should preferably have been proven with biopsy and bone scan and accompanied by medical bone-related laboratory investigations, which in our series was not performed on all patients, as most of the cases predate the current best practice guideline.^19^ Therefore, there is a chance that some of our cases may have been polyostotic or involved endocrine disorders. Last, the treatment of FD is a multidisciplinary endeavor in which systemic treatment with bisphosphonates and monoclonal antibodies (denosumab) complements the surgical management. However, this was beyond the scope of this surgical study.

## Conclusion

The treatment of proximal femoral MFD presents with specific challenges, particularly in children. All treatment decisions should be made on an individual basis, as each lesion has its characteristics and risks. In selected cases, such as those with thick cortices, with low fracture and deformity risks at diagnosis, watchful waiting may be a viable option. If treatment is required, internal fixation should be considered to prevent fractures and deformity. Patients with fracture at diagnosis have a higher risk of failure in follow-up (i.e., (re)fractures, (re)procedures).

The goal of MFD treatment is not eradication of the lesion, but prevention of complications and alleviation of symptoms in the least invasive manner possible. In this respect, internal fixation is a logical treatment option, while curettage with cancellous bone grafting, percutaneous bone substitute injections, or strut grafts should be used with hesitance.

## Supplemental Material

sj-jpg-2-cho-10.1177_18632521251355884 – Supplemental material for Surgical treatment of monostotic fibrous dysplasia of the proximal femur in children and adolescents: Observational European Paediatric Orthopaedic Society multicenter study Supplemental material, sj-jpg-2-cho-10.1177_18632521251355884 for Surgical treatment of monostotic fibrous dysplasia of the proximal femur in children and adolescents: Observational European Paediatric Orthopaedic Society multicenter study by Thomas PG van Geloven, Pieter Bas de Witte, Minna K Laitinen, Domenico A Campanacci, Kevin Döring, Dietmar Dammerer, Mohamed K Mesregah, Natasja M Appelman-Dijkstra, Mikko Haara, Giovanni Beltrami, Gerhard M Hobusch, Tanja Kraus, Sebastian Farr, Camilo Soto-Montoya, Manuel R Medellin Rincon, Javeria Saeed, Phillipp T Funovics, Lizz van der Heijden and Michiel AJ van de Sande in Journal of Children’s Orthopaedics

sj-pdf-1-cho-10.1177_18632521251355884 – Supplemental material for Surgical treatment of monostotic fibrous dysplasia of the proximal femur in children and adolescents: Observational European Paediatric Orthopaedic Society multicenter study Supplemental material, sj-pdf-1-cho-10.1177_18632521251355884 for Surgical treatment of monostotic fibrous dysplasia of the proximal femur in children and adolescents: Observational European Paediatric Orthopaedic Society multicenter study by Thomas PG van Geloven, Pieter Bas de Witte, Minna K Laitinen, Domenico A Campanacci, Kevin Döring, Dietmar Dammerer, Mohamed K Mesregah, Natasja M Appelman-Dijkstra, Mikko Haara, Giovanni Beltrami, Gerhard M Hobusch, Tanja Kraus, Sebastian Farr, Camilo Soto-Montoya, Manuel R Medellin Rincon, Javeria Saeed, Phillipp T Funovics, Lizz van der Heijden and Michiel AJ van de Sande in Journal of Children’s Orthopaedics
