# Supplementary figures and images for: Surgical treatment of monostotic fibrous dysplasia of the proximal femur in children and adolescents: Observational European Paediatric Orthopaedic Society multicenter study
Source: J Child Orthop. 2025 Jul 24:18632521251355884. Online ahead of print. doi: 10.1177/18632521251355884 (PMC12289611; doi:10.1177/18632521251355884)

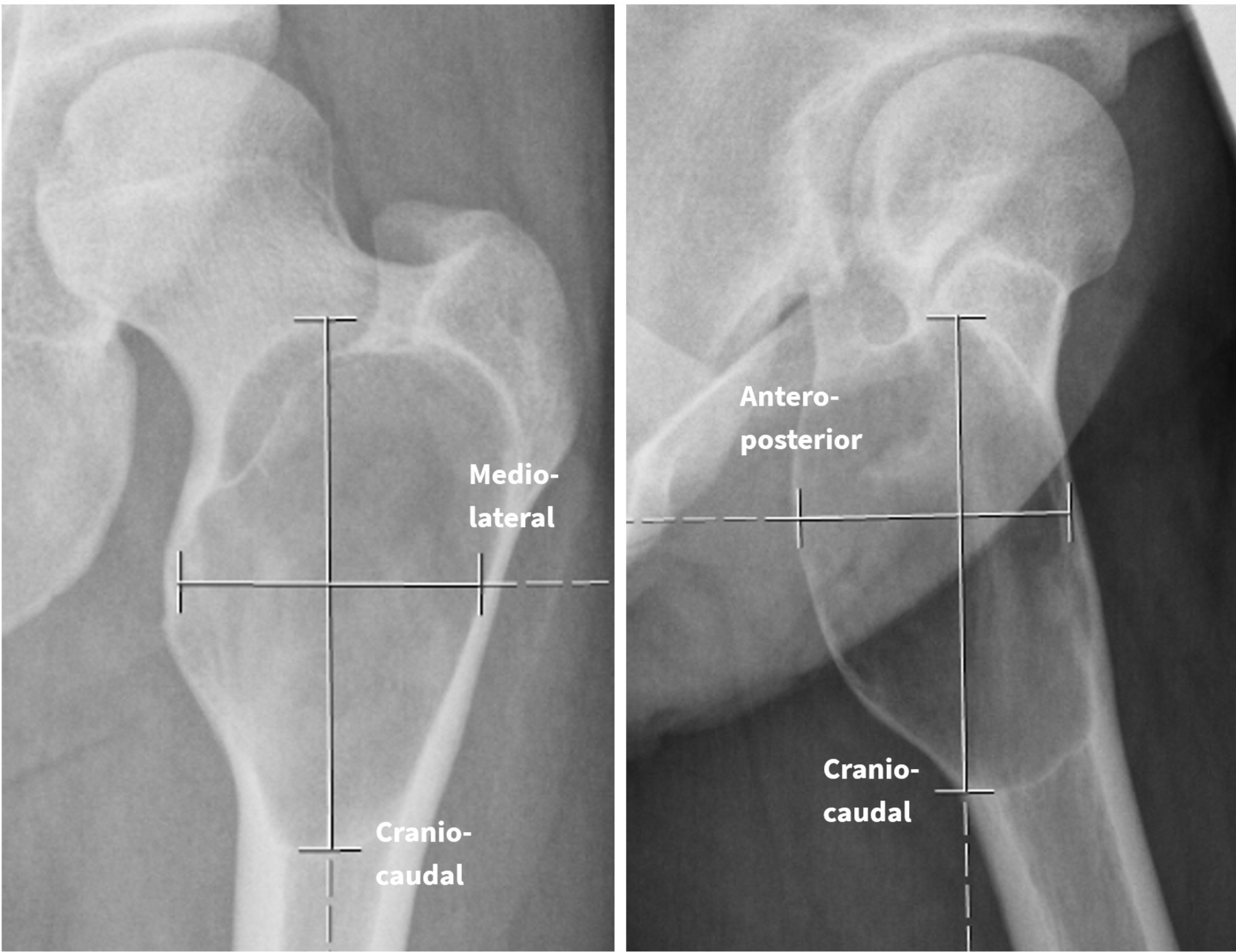

Supplement: sj-jpg-2-cho-10.1177_18632521251355884 – Supplemental material for Surgical treatment of monostotic fibrous dysplasia of the proximal femur in children and adolescents: Observational European Paediatric Orthopaedic Society multicenter study [file sj-jpg-2-cho-10.1177_18632521251355884.jpg]
